# ISAR Imaging Based on the Wideband Hyperbolic Frequency-Modulation Waveform

**DOI:** 10.3390/s150923188

**Published:** 2015-09-15

**Authors:** Wei Zhou, Chun-mao Yeh, Kan Jin, Jian Yang, Yao-bin Lu

**Affiliations:** 1 Department of Electronic Engineering, Tsinghua University, Beijing 100084, China; E-Mails: kevincabbage@sina.com (K.J.); yangjian_ee@mail.tsinghua.edu.cn (J.Y.); 2 Beijing Institute of Radio Measurement, Beijing 100039, China; E-Mails: chunmaoyeh@gmail.com (C.Y.); luyaobing65@163.com (Y.L.)

**Keywords:** inverse synthetic aperture radar (ISAR), hyperbolic frequency modulated (HFM), linear frequency modulated (LFM), Doppler invariant, pulse compression

## Abstract

The hyperbolic frequency-modulated (HFM) waveform has an inherent Doppler-invariant property. It is more conducive than the conventional linear frequency-modulated (LFM) waveform to high speed moving target imaging. In order to apply the HFM waveform to existing inverse synthetic aperture radar (ISAR) imaging systems, a new pulse compression algorithm is proposed. First, the received HFM echoes are demodulated with the transmitted signal, which is called “decurve” in this paper. By this operation, the bandwidth of the demodulated echoes is effectively reduced and can be processed by the existing narrow-band receiver. Then, the phase of the decurved HFM echoes is analyzed, and thus, the pulse compression is accomplished by space-variant phase compensation. In addition, the space-variant phase compensation is realized by resampling and fast Fourier transform (FFT) with high computational efficiency. Finally, numerical results illustrate the effectiveness of the proposed method.

## 1. Introduction

A radar typically transmits waveforms with a large time-bandwidth product (TBP), in order to achieve a long-range detection ability with high resolution. The conventional linear frequency-modulated (LFM) waveform has excellent pulse compression performance, and its range resolution is inversely proportional to the signal bandwidth [1]. However, when the relative velocity between a radar and a target is relatively high, the widely-used “stop-and-go” assumption is no longer valid. In this case, the received LFM echoes may be Doppler distorted, and the output of the matched filter will be degraded significantly [2]. Several methods have been proposed to compensate for the high speed influences on wideband LFM echoes [3,4,5]. Although these methods are proven to be effective to some extent, they are complex to implement or suffer from heavy computational burden. This problem becomes more complicated when two or more high speed moving targets with different velocities exist in a single radar beam, such as a satellite and the nearby space debris. In order to get a focused high resolution range profile (HRRP) for each target, it is necessary to segment the targets from the defocused range profiles and to compensate their high speed influences separately, which is very difficult to implement for practical systems. Therefore, the Doppler-tolerant waveform with the minimum signal loss under different Doppler environments is always desired. The hyperbolic frequency-modulated (HFM) waveform, also be called the linear period-modulated (LPM) waveform [1], meets the optimum frequency modulation law and has an inherent Doppler-invariant property [6,7]. Nowadays, the HFM waveforms have been widely used in radar and sonar systems [8,9,10].

Moreover, when two closely-placed radars use the same frequency band, it is proven in [10] that “the radio frequency interference (RFI) can be significantly reduced by using LFM in one radar and HFM in the other”. Additionally, “the improvement increases with time-bandwidth product and also with the ratio of the bandwidth to the starting frequency” [10]. Therefore, the application of the wideband HFM waveform also has the potential to improve the bandwidth efficiency in multi-radar systems.

The pulse compression of the HFM waveform is conventionally achieved through matched filtering [1,6], which needs a sampling frequency higher than the bandwidth according to the conventional Nyquist sampling theory. When the TBP of the transmitted waveform gets larger, there will be a significant increase for the storage and computational load. In the application of wideband LFM waveforms, this problem is solved by using the dechirping method to reduce the sampling frequency [11,12]. In order to apply the HFM waveform to the existing radar systems, which send the LFM waveform and use the dechirping method for pulse compression, a new pulse compression algorithm for the HFM waveform is proposed. The bandwidth of received signals is effectively reduced by demodulating with the transmitted signal. Since both the received and reference signals are nonlinear frequency modulated, we call this method “decurve” in this paper. Then, the demodulated echoes can be processed by the existing narrow-band receiver, which is very similar to the dechirp processing. However, in this case, the received echoes are still nonlinear frequency-modulated signals after decurve processing, and the pulse compression cannot be achieved directly by the fast Fourier transform (FFT). By constructing a phase matching (PM) function in this paper, the non-linear phase in the received echoes is effectively compensated, and the pulse compression for the HFM waveform is finally realized. To make the computation more efficient, a method combining resampling and FFT is also proposed for the pulse compression after decurve processing.

The manuscript is organized as follows. Section 2 briefly summarizes the Doppler property of the LFM waveform. Section 3 presents the ISAR processing scheme for high speed moving targets based on the HFM waveform. Additionally, the proposed decurving method for wideband HFM radar echoes is emphasized. Finally, numerical simulations demonstrate the effectiveness of the proposed method in Section 4.

## 2. Doppler Property of the LFM Waveform

The well-known LFM waveform is very convenient in many applications for its high performance in pulse compression. However, when signals with large TBP are used for high speed moving target imaging, the LFM waveform may lose many of its advantages according to the radar echo model.

The transmitted wideband LFM waveform is given by:

(1) $$s_{\mathrm{lt}}\left(t\right)=a\left(\frac{t}{T_{p}}\right)\exp\left[j2\pi \left(f_{c}t+\frac{1}{2}\gamma t^{2}\right)\right]$$

where $T_{p}$ is the pulse width, *t* is the time variable, $f_{c}$ is the carrier frequency, $\gamma =B/T_{p}$ is the linear frequency modulation coefficient and *B* is the bandwidth. The function $a\left(x\right)$ in Equation (1) is the rectangular window function, which equals one when $\left|x\right|\leq 0.5$ and zero otherwise.

For a high speed moving target, the conventional stop-go assumption is not valid. The returned signal from the *n*-th scattering point on the target may be expressed as [1]:

(2) $$s_{\mathrm{lr}}\left(t\right)=\sigma_{n0}s_{\mathrm{lt}}\left(\beta \left(t-\tau_{n0}\right)\right)$$

where $\tau_{n0}=2R_{n}/c$ is the propagation time, $R_{n}$ is the distance between the radar and the scattering point, *c* is the speed of light, $\sigma_{n0}$ is the reflection coefficient, *β* is the Doppler factor given by $\beta =\left(c-\upsilon \right)/\left(c+\upsilon \right)$ and *υ* is the relative velocity between the radar and the target, which is assumed to be constant during the time of a single pulse width in this paper. Substituting Equation (1) into Equation (2), the returned signal from the *n*-th scattering point on the target will be:

(3) $$s_{\mathrm{lr}}\left(t\right)=\sigma_{n0}a\left(\frac{\beta \left(t-\tau_{n0}\right)}{T_{p}}\right)\exp\left[j2\pi f_{c}\beta \left(t-\tau_{n0}\right)+j\pi \gamma \beta^{2}{\left(t-\tau_{n0}\right)}^{2}\right]$$

**Figure 1:**
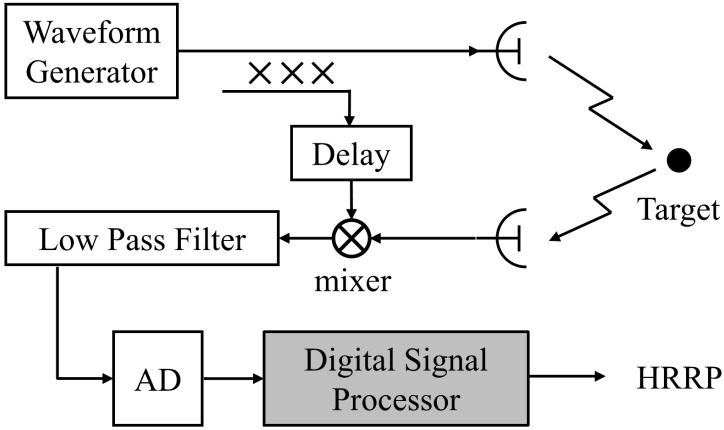
The existing LFM radar configuration.

Limited by the processing capacity of the receiver, the wideband echoes are usually processed by the dechirping method to reduce the sampling rate [11], as shown in Figure 1. The received echoes are first demodulated with the transmitted signal, followed by a low pass filter to get the baseband signal. Then, the baseband signal is in-phase and quadrature (I/Q) sampled and further processed to generate the HRRP.

The reference signal for dechirp processing is set to be:

(4) $$s_{\mathrm{lref}}\left(t\right)=a\left(\frac{t-\tau_{m}}{T_{p}}\right)\exp\left[j2\pi f_{c}\left(t-\tau_{m}\right)+j\pi \gamma {\left(t-\tau_{m}\right)}^{2}\right]$$

where $\tau_{m}=2R_{m}/c$ and $R_{m}$ is the reference distance.

The output of the dechirp processing may be expressed as:

(5) $$s_{\mathrm{ld}}\left(t\right)=s_{\mathrm{lr}}\left(t\right)s_{\mathrm{lref}}^{*}=\sigma_{n0}a\left(\frac{t-\tau_{m}}{T_{p}}\right)\exp\left[j\left(\phi_{0}+\phi_{1}+\phi_{2}+\phi_{3}\right)\right]$$

(6) $$\phi_{0}=2\pi f_{c}\left(\tau_{m}-\beta \tau_{n0}\right)$$

(7) $$\phi_{1}=\pi \gamma \left(\beta^{2}\tau_{n0}^{2}-\tau_{m}^{2}\right)$$

(8) $$\phi_{2}=2\pi \left[\left(\beta -1\right)f_{c}+\gamma \left(\tau_{m}-\beta \tau_{n0}\right)\right]t$$

(9) $$\phi_{3}=\pi \gamma \left(\beta^{2}-1\right)t^{2}$$

where $s_{\mathrm{lref}}^{*}\left(t\right)$ is the conjugate of $s_{\mathrm{ref}}\left(t\right)$, $\phi_{0}$ is the phase term related to the scattering point’s initial position and the Doppler factor, $\phi_{1}$ is the residual video phase (RVP), $\phi_{2}$ is the single frequency component, which will decide the position of the scattering point on the range profile, and the second order phase term $\phi_{3}$ is induced by the Doppler modulation, which will cause a range migration that must be accounted for.

We can see that $\phi_{3}$ is space invariant and is just related to the linear frequency modulation coefficient and the Doppler factor. When the target’s velocity is slow, $\phi_{3}$ is negligible, and the pulse compression can be achieved by simple FFT. For high speed moving targets, two kinds of methods can be used for the compensating of $\phi_{3}$. The first is to make use of the target tracking information. The target velocity can be estimated via curve fitting over multiple reference range measurements. Then, a second-order phase compensation function is constructed according to $\phi_{3}$. This method is simple, but is less accurate, since large errors exist in the reference range measurements [13]. The other kind is to estimate the Doppler frequency modulation rate of each range image [3,4,5]. However, these methods are complex in computation, for the time-consuming parameter searching algorithm should be taken for each range image.

## 3. HFM-Based ISAR Imaging

In this section, we first briefly summarize the Doppler-invariant property of the HFM waveform. Then, the proposed decurve processing for the HFM waveform is derived in detail. Additionally, the pulse compression can be achieved without any information about the target’s velocity. There is no necessity for velocity compensation before the ISAR imaging. Therefore, the traditional autofocusing methods can be directly used for the range alignment and translation phase compensation with high efficiency.

### 3.1. Doppler-Invariant Property of the HFM Waveform

The HFM waveform is expressed as:

(10) $$s_{\mathrm{ht}}\left(t\right)=a\left(\frac{t}{T_{p}}\right)\exp\left[j\frac{2\pi }{b}\ln\left(1-\frac{B}{f_{c}}\frac{t}{T_{p}}\right)\right]$$

where *b* denotes the hyperbolic frequency modulation coefficient. If we set the starting frequency as $f_{L}=f_{c}-B/2$ and ending frequency as $f_{H}=f_{c}+B/2$, the frequency modulation coefficient will be $b=-B/\left(f_{L}f_{H}T_{p}\right)$. The instantaneous frequency of the HFM waveform in Equation (10) is then given by:

(11) $$f_{ht}\left(t\right)=\frac{f_{H}f_{L}}{f_{c}-\gamma t},\;\;\;\left|t\right|\leq \frac{T_{p}}{2}$$

**Figure 2:**
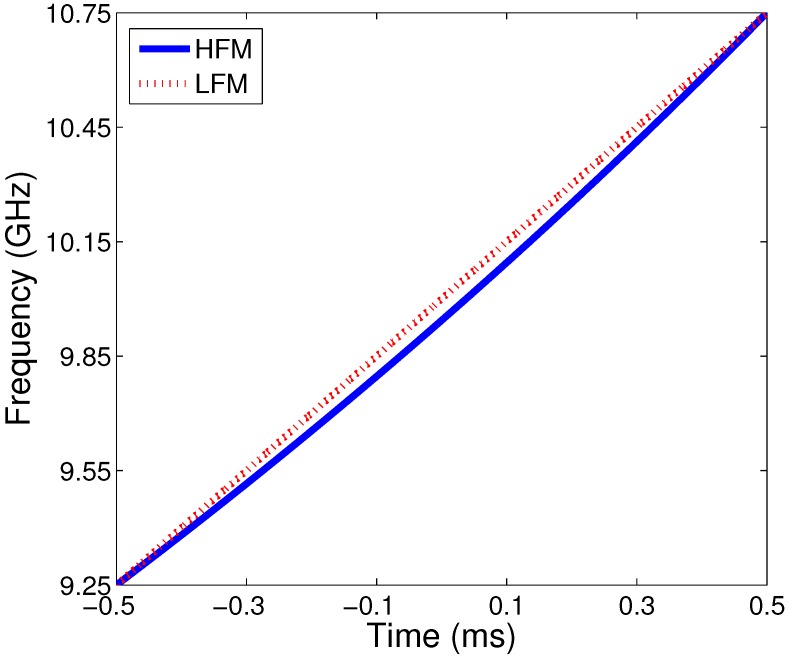
The instantaneous frequency of HFM and LFM waveforms, B=1.5 GHz, $f_{c}=10$ GHz, $T_{p}=1.0$ ms.

The instantaneous frequency of a typical HFM waveform is shown in Figure 2.

Similar to Equation (2), we can get the returned signal from the scattering point as:

(12) $$\begin{array}{c} s_{hr}\left(t\right)=\sigma_{n0}s_{ht}\left(\beta \left(t-\tau_{n0}\right)\right)\\ \;\;\;\;\;\;\;\;\;\;=\sigma_{n0}a\left(\frac{\beta \left(t-\tau_{n0}\right)}{T_{p}}\right)\exp\left(j\varphi_{0}\right)\exp\left[j\frac{2\pi }{b}\ln\left(1-\frac{\gamma }{f_{c}}\left(t-\tau_{n0}-\tau_{\upsilon }\right)\right)\right] \end{array}$$

where $\varphi_{0}=2\pi \ln\beta /b$ is a constant value, and $\tau_{\upsilon }$ is a time bias caused by the Doppler effect, given by:

(13) $$\tau_{\upsilon }=\frac{2\upsilon }{c-\upsilon }\frac{f_{c}T_{p}}{B}$$

Ignoring the stretch of the rectangle window, the received signal in Equation (12) takes the following form:

(14) $$s_{hr}\left(t\right)=\sigma_{n}a\left(\frac{t-\tau_{n}}{T_{p}}\right)\exp\left[j\frac{2\pi }{b}\ln\left(1-\frac{\gamma \left(t-\tau_{n}\right)}{f_{c}}\right)\right]$$

where $\tau_{n}=\tau_{n0}+\tau_{\upsilon }$ and $\sigma_{n}=\sigma_{n0}\exp\left(j\varphi_{0}\right)$. Equation (14) indicates that the Doppler effect of HFM waveform can be simplified as a range bias combined with a constant phase shift. If the instantaneous frequency of $s_{hr}\left(t\right)$ is $f_{hr}\left(t\right)$, then we will get:

(15) $$f_{hr}\left(t\right)=f_{ht}\left(t-\tau_{n}\right)$$

Equation (15) means that the HFM waveform meets the optimum frequency modulation law [1], which can also explain that the HFM waveform is Doppler invariant.

### 3.2. Decurve Processing with the Existing Configuration

The decurve processing is proposed just based on the radar configuration in Figure 1. Therefore, the HFM waveform can be applied to the existing LFM radar systems just by replacing the LFM waveform generator with the HFM waveform generator, and the other parts of the hardware configuration can stay the same.

For HFM echoes, the reference signal for decurve processing is set to be:

(16) $$s_{href}\left(t\right)=a\left(\frac{t-\tau_{m}}{T_{p}}\right)\exp\left[j\frac{2\pi }{b}\ln\left(1-\frac{\gamma \left(t-\tau_{m}\right)}{f_{c}}\right)\right]$$

The output of the proposed decurve processing method in this paper can be expressed as:

(17) $$\begin{array}{ccc} s_{hd}\left(t\right) & = & s_{hr}\left(t\right)s_{href}^{*}\left(t\right)\\ & = & \sigma_{n}a\left(\frac{t-\tau_{m}}{T_{p}}\right)\exp\left\{j\frac{2\pi }{b}\ln\left[1-\frac{\gamma \tau_{mn}}{f_{c}-\gamma \left(t-\tau_{m}\right)}\right]\right\} \end{array}$$

where $\tau_{mn}=\tau_{m}-\tau_{n}$, and the phase term of Equation (17) can be expanded by (refer to Equation (A20) in the Appendix):

(18) $$\begin{array}{c} \varphi \left(t\right)=\frac{2\pi }{b}\ln\left[1-\frac{\gamma \tau_{mn}}{f_{c}-\gamma \left(t-\tau_{m}\right)}\right]\\ \;\;\;\;\;\;\;\;\approx 2\pi Hf_{c}\tau_{mn}+2\pi f_{n}\frac{t-\tau_{m}}{1-\frac{\gamma }{f_{c}}\left(t-\tau_{m}\right)} \end{array}$$

where $H=f_{L}f_{H}/f_{c}^{2}$ is a constant value and $f_{n}=H\gamma \tau_{mn}$ is the center frequency of $s_{hd}\left(t\right)$. The effective bandwidth of the demodulated baseband signal is (refer to Equation (A22) in the Appendix):

(19) $$B_{d}\leq B_{r}=H\gamma \frac{2r_{\mathrm{wid}}}{c}\frac{1}{{\left(1-\frac{B}{2f_{c}}\right)}^{2}}$$

where $r_{wid}$ is the maximum width of the imaging scene. We can see from Equation (19) that a sampling rate higher than $B_{r}$ will be high enough with no loss. From Equation (18), we can see that the signals after decurve processing contain a nonlinear frequency-modulated component, which is related to $\tau_{mn}$ and is space variant. Therefore, the pulse compression cannot be achieved by simple FFT. In order to compensate the space-variant phase term in Equation (18) and to get the focused HRRP, we construct the space-variant phase matching (PM) function as:

(20) $$s_{k}\left(t\right)=\exp\left[-j2\pi f_{k}\frac{t-\tau_{m}}{1-\frac{\gamma }{f_{c}}\left(t-\tau_{m}\right)}\right]$$

where *k* is the sampling index and the total number of sampling points is *K* ; then, the output of pulse compression at frequency $f_{k}$ will be:

(21) $$\begin{array}{c} h\left(f_{k}\right)=\frac{1}{K}\sum_{l=0}^{K-1}s_{\mathrm{hd}}\left(t_{l}\right)s_{k}\left(t_{l}\right)\\ \;\;\;\;\;\;\;\;=\frac{1}{K}\sum_{l=0}^{K-1}\sigma_{n}a\left(\frac{t_{l}-\tau_{m}}{T_{p}}\right)\exp\left\{j2\pi Hf_{c}\tau_{mn}+j2\pi f_{n}\frac{t-\tau_{m}}{1-\frac{\gamma }{f_{c}}\left(t-\tau_{m}\right)}-j2\pi f_{k}\frac{t_{l}-\tau_{m}}{1-\frac{\gamma }{f_{c}}\left(t_{l}-\tau_{m}\right)}\right\}\\ \;\;\;\;\;\;\;\;=\frac{1}{K}\sum_{l=0}^{K-1}\sigma_{n}a\left(\frac{t_{l}-\tau_{m}}{T_{p}}\right)\exp\left\{j2\pi Hf_{c}\tau_{mn}+j2\pi \left(f_{n}-f_{k}\right)\left(t_{l}-\tau_{m}\right)\left[1+\sum_{i=1}^{\infty }\frac{\gamma^{i}}{f_{c}^{i}}{\left(t_{l}-\tau_{m}\right)}^{i}\right]\right\} \end{array}$$

Since:

(22) $$\frac{1}{K}\sum_{l=0}^{K-1}a\left(\frac{t_{l}-\tau_{m}}{T_{p}}\right)\exp\left[j2\pi \left(f_{n}-f_{k}\right)\left(t_{l}-\tau_{m}\right)\right]=\mathrm{sinc}\left(\frac{f_{k}-f_{n}}{1/T_{p}}\right)$$

and:

(23) $$\frac{1}{K}\sum_{l=0}^{K-1}a\left(\frac{t_{l}-\tau_{m}}{T_{p}}\right)\exp\left[j2\pi \left(f_{n}-f_{k}\right)\frac{\gamma^{i}}{f_{c}^{i}}{\left(t_{l}-\tau_{m}\right)}^{i+1}\right]\approx 0,\;\;i=1,2,\cdots$$

We can get that:

(24) $$h\left(f_{k}\right)\approx \sigma_{n}\mathrm{sinc}\left(\frac{f_{k}-f_{n}}{\Delta f}\right)\exp\left(j2\pi Hf_{c}\tau_{mn}\right)$$

where $\Delta f=1/T_{p}$ is the frequency resolution cell.

From Equation (21), we can see that the PM method needs $K^{2}$ times of multiplication for range imaging. In order to reduce the computational cost, a more efficient pulse compression method based on resampling combined with FFT is also proposed in this paper. The resampled time is given by:

(25) $$t^{\prime }=\frac{t-\tau_{m}}{1-\frac{\gamma }{f_{c}}\left(t-\tau_{m}\right)}$$

Substituting Equation (25) into Equations (17) and (18) yields:

(26) $$s_{hd}\left(t^{\prime }\right)\approx \sigma_{n}a\left(\frac{t^{\prime \prime }}{T_{p}}\right)\exp\left[j2\pi \left(Hf_{c}\tau_{mn}+f_{n}t^{\prime }\right)\right]$$

where $t^{\prime \prime }=t^{\prime }/\left(1+\gamma t^{\prime }/f_{c}\right)$. Ignoring the stretch of the rectangle window in Equation (23), the pulse compression can be achieved directly using FFT.

**Figure 3:**
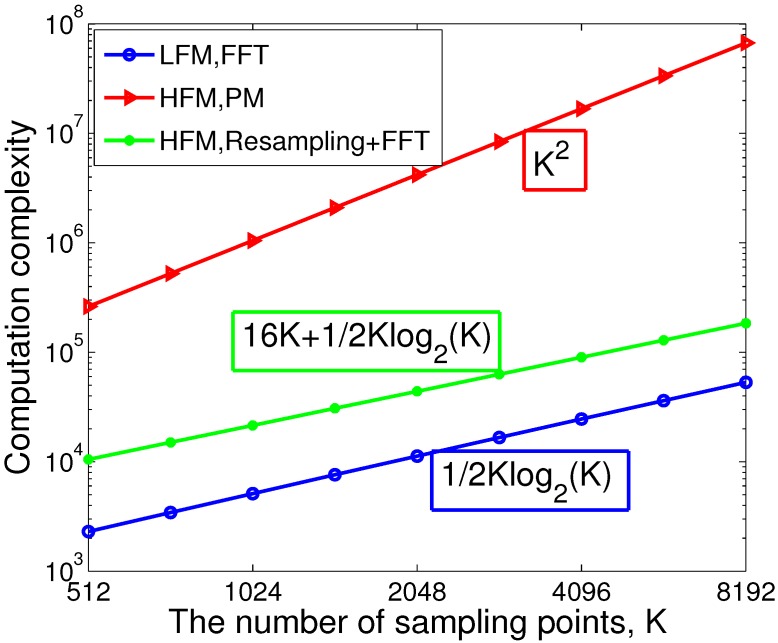
The computational complexity of the proposed pulse compression method for the HFM and LFM waveforms.

The resampling operation can be realized by discrete sinc interpolation [14], and the coefficients of the sinc interpolation for a given system can be computed and stored in advance. Therefore, the computational complexity will be $O(LK+1/2K\log_{2}K)$ if the length of sinc interpolation is *L*. In our test, the sinc interpolation with L=16 is precise enough, and the computation cost of the proposed pulse compression method is shown in Figure 3, while the computation complexity of FFT ($1/2K\log_{2}K$) is given for comparison. Although the computation complexity of the proposed method is a little higher than FFT, there is no pressure for real-time realization based on the present high-performance digital processor. In addition, when the radar transmits the LFM waveform with large TBP, the target velocity should be estimated to compensate the Doppler effect, which will introduce additional computational load.

## 4. Experimental Section

In this section, we first examine the RFI between the LFM and HFM waveforms. Then, we will provide several numerical experiments of one- and two-dimensional imaging based on both the LFM and HFM waveform and compare their performance under a serious Doppler effect.

### 4.1. RFI between LFM and HFM

**Figure 4:**
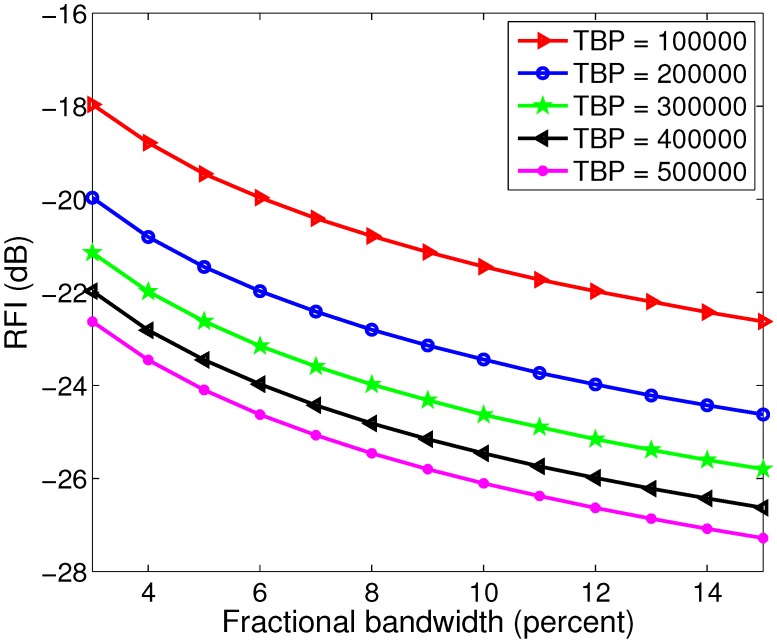
The radio frequency interference (RFI) between LFM and HFM with various fractional bandwidths and the time-bandwidth product (TBP).

The RFI between two nearby radars in the same band can be very slow by using LFM in one radar and HFM in the other radar. Since some related results can be directly found in [10], we just consider the RFI when the decurving method is used for pulse compression. Suppose that in the first case, the transmitted waveform is LFM, and the maximum value of the HRRP after decurve processing is $h_{1}$. In the second case, the transmitted waveform is HFM, and the maximum value of the HRRP is $h_{2}$. Then, we define the degree of the RFI as:

(27) $$RFI=20\log_{10}\left(\frac{h_{1}}{h_{2}}\right)$$

which is shown in Figure 4. The fractional bandwidth in Figure 4 is defined by the ratio of the bandwidth to the starting frequency. We can see that the RFI between the LFM and HFM waveforms decreases with the TBP and the fractional bandwidth of the transmitted waveform. For waveforms with TBP > 100,000, the RFI is lower than −18 dB, which is almost negligible in practice. This property is very useful in multi-radar systems, where the RFI between radars must be taken into account.

### 4.2. HRRPs with the LFM and HFM Waveforms

**Figure 5:**
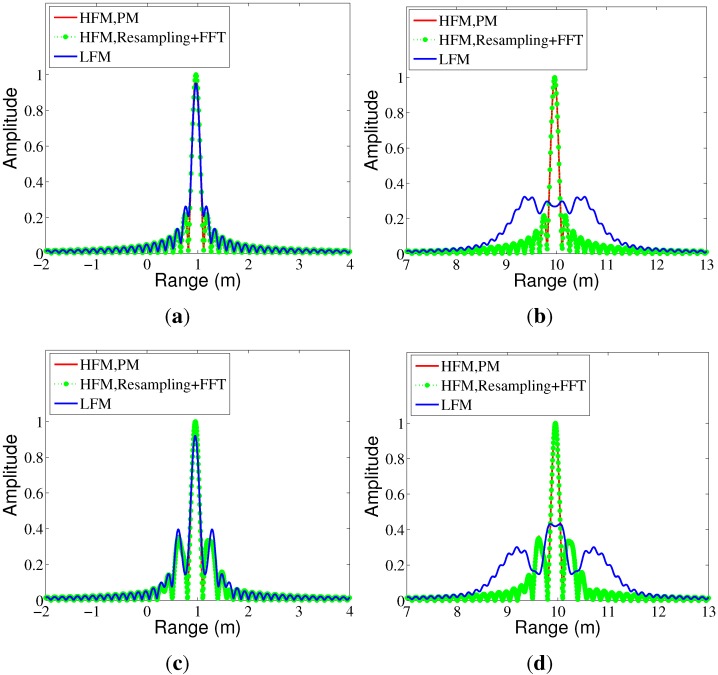
The high resolution range profile (HRRP) of a single point target. (**a**) Ideal waveform, υ=100 m/s; (**b**) ideal waveform, υ=1000 m/s; (**c**) with frequency errors, υ=100 m/s; (**d**) with frequency errors, υ=1000 m/s.

In order to compare the Doppler property of the LFM and HFM waveforms more clearly, a target with only one point is used, and the pulse compression results are shown in Figure 5. For both LFM and HFM, the duration of the transmitted pulse is 1 ms; the carrier frequency is 10 GHz; the bandwidth is 1 GHz; and the pulse compression is achieved without any windowing operation. The sampling frequency after decurve or dechirp processing is set to be 10 MHz. The performance of the pulse compression is evaluated in terms of the peak sidelobe ratio (PSLR) and integrated sidelobe ratio (ISLR). We can see that when the target velocity is 100 m/s, the HRRP of both LFM and HFM radar echoes have very high range resolution, as shown in Figure 5a. When the target velocity is 1000 m/s, the LFM suffers from obvious Doppler distortion, and the HRRP of the LFM waveform is expanded after pulse compression, as shown in Figure 5b. In this case, the PSLR and ISLR are not calculated, for there is no clear difference between the mainlobe and sidelobe. However, the PSLR and ISLR of the HFM waveform stay almost unchanged when the target velocity varies from 100 m/s to 1000 m/s, as shown in Table 1. As proven in [15], the LFM waveform has a Doppler width proportional to the reciprocal of the TBP; while the signal loss of HFM radar echoes under different target velocities is almost negligible and the HRRPs of the HFM echoes stay undistorted. We can also see from Figure 5 that the efficient pulse compression method based on resampling and FFT has almost the same precision compared to the PM method, while the computation is largely reduced.

In this paper, the pulse compression with unideal waveform errors is also concerned, and the instantaneous frequency error is modeled by the cosine function as [16]:

(28) $$f_{e}\left(t\right)=LB\cos\left(2\pi t/T_{e}\right)$$

where *L* can be treated as the linearity coefficient of the frequency modulation, and $T_{e}$ is the period of the frequency error. The same frequency errors are added to the LFM and HFM waveforms, and the pulse compression results with $L=10^{-4}$ and $T_{e}=0.5$ ms are shown in Figure 5c,d. The relative PSLR and ISLR are shown in Table 1. We can see that the unideal waveform errors may cause a higher sidelobe and decrease the pulse compression performance for both the LFM and HFM waveforms. The HFM waveform achieves better pulse compression performance based on its Doppler-invariant property. Additionally, the pulse compression of the LFM waveform is damaged by both the target’s Doppler and the frequency errors.

**Table 1:** Peak sidelobe ratio (PSLR) and integrated sidelobe ratio (ISLR) with different velocities.

Waveform	Velocity (m/s)	Ideal Waveform		With Frequency Errors	
		PSLR (dB)	ISLR (dB)	PSLR (dB)	ISLR (dB)
LFM	100	−11.22	−7.91	−7.32	−3.88
	1000	-	-	-	-
HFM	100	−13.27	−9.60	−9.13	−4.49
	1000	−13.27	−9.60	−9.13	−4.49

### 4.3. ISAR Imaging Simulation

**Figure 6:**
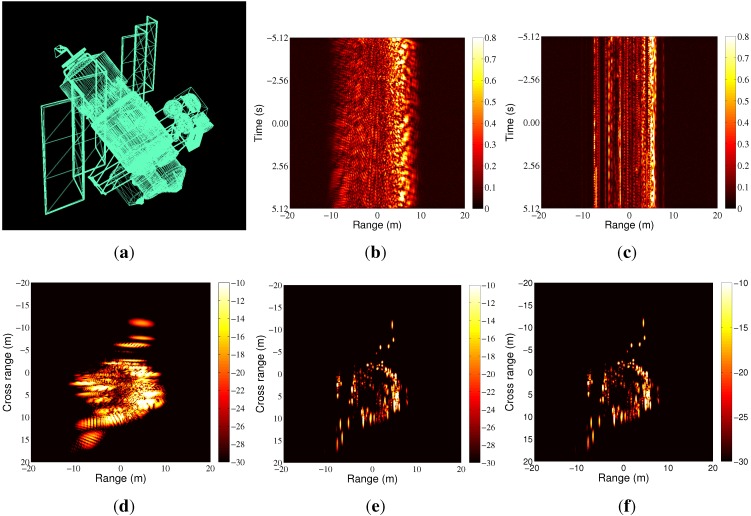
ISAR imaging simulation. (**a**) 3D satellite model; (**b**) LFM, the simulated range profiles; (**c**) HFM, the simulated range profiles; (**d**) LFM, the range-Doppler (RD) imaging result without velocity compensation; (**e**) LFM, the RD imaging result with velocity compensation by integrated cubic phase function (ICPF); (**f**) HFM, the RD imaging result.

In this case, a three-dimensional (3D) satellite model is used, as shown in Figure 6a. The length of the target’s main body is about 19.5 m, and the width of the target including the solar panels is about 31.2 m. In our simulation, the high-frequency electromagnetic scattering is obtained through the physical optics (PO) method. The target is located 500 km away from the radar and moves along a straight line with a speed υ=5000 m/s relative to the radar. For both LFM and HFM, the duration of the transmitted pulse is 1 ms; the carrier frequency is 10 GHz; and the bandwidth is 1 GHz. The data collection time is set to be 10.24 s, and the simulated ISAR system transmits 100 chirp signals per second. During this time interval, the target’s equivalent rotation angle is 2.47${}^{\circ }$, and the resulting cross-range resolution is about 0.35 m. All of the simulations are taken on a MATLAB R2012a platform with a Core i5 CPU.

**Figure 7:**
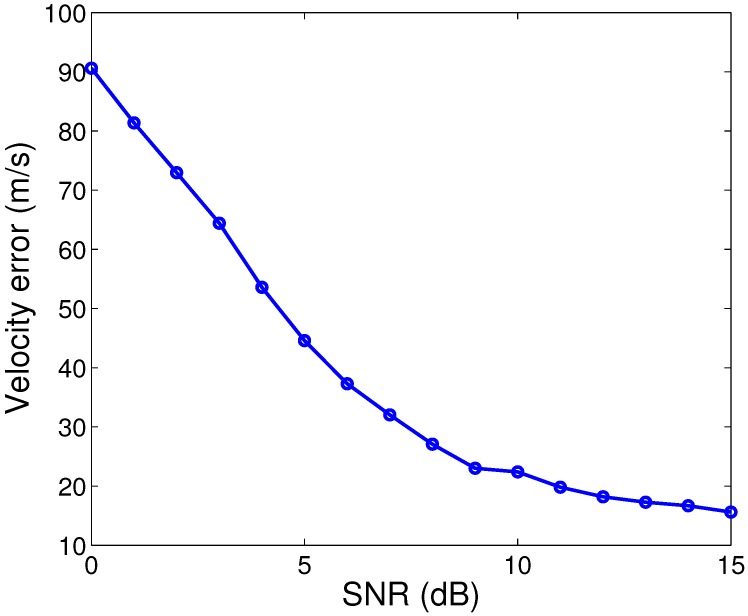
The estimated velocity errors with the ICPF method under different SNRs.

The simulated LFM and HFM range profiles after range alignment are shown in Figure 6b,c. The traditional translation motion compensation (TMC), including range alignment and phase compensation, is realized by the accumulated maximum correlation method [17] and the modified Doppler centroid tracking (MDCT) method [18]. Both of these methods have relatively low computational load and are robust under noise environment. The range-Doppler (RD) imaging result of the LFM radar echoes without velocity compensation is shown in Figure 6d. It is obvious to see that the received radar echoes are Doppler distorted, resulting in blurring in the range direction. To compensate the Doppler effect, the method based on the integrated cubic phase function (ICPF) [5] is used, and the relative RD image is shown in Figure 6e. The focusing quality is measured by the image entropy and image contrast, as shown in Table 2. We can see that the image quality has been improved greatly after velocity compensation. However, the ICPF estimates the target velocity based on a line search method, which is time consuming. The computational complexity of the ICPF in [5] is about $O(MNK^{2})$, where *M* is the number of transmitted waveforms, *K* is the number of sampling points in the range direction and *N* is the number of searched velocities. Additionally, in this simulation, the initial error of the target velocity measured from the narrowband tracking system is set to be 250 m/s, and the search step is 5 m/s, resulting in N=100. The other parameters are set as M=1024, K=10,000 according to the radar signal parameters. The total time cost of the TMC including velocity compensation with ICPF is 280.13 s, as shown in Table 2. Moreover, the ICPF-based method is sensitive to noise to some extent, and the performance gets worse when the SNR gets lower, as shown in Figure 7. In our simulation, the simulated SNR is about 10 dB, and the average error of the estimated target velocity is about 24.4 m/s. When the HFM is used, no prior information or estimation about the target velocity is needed to obtain focused ISAR images. The RD image of the HFM radar echoes is shown in Figure 6f, and the time cost of TMC is 3.22 s. From Table 2, we can see that although the focusing quality between Figure 6e,f is small, the processing time is much less when HFM is used, which is very beneficial for real-time imaging of high speed moving targets.

**Table 2:** ISAR imaging simulation results.

Waveform	Velocity Compensation	Time Cost (s)	Image Entropy	Image Contrast
LFM	no	3.25	12.71	18.50
	ICPF	280.13	10.53	42.91
HFM	no	3.22	10.50	43.08

## 5. Conclusions

The good performance of the HFM under a large Doppler effect makes it very useful in high speed moving target detection and imaging. Additionally, the application of the wideband HFM waveform also has the potential to improve the bandwidth efficiency in multi-radar systems, for the low RFI between the LFM and HFM waveforms. In this paper, a new pulse compression method has been proposed for the HFM waveforms. The bandwidth of the demodulated echoes was largely reduced through the decurve processing, and then, both the phase matching method and the efficient method combing resampling and FFT were used to obtain the focused HRRP. For the similarity between the decurve and dechirp processing, the HFM waveform was applied to the existing imaging radar systems that are more convenient with no change in the receiving channel. Both one- and two-dimensional radar imaging results have been taken to prove the correctness of the proposed method. Moreover, when the wideband HFM waveform is applied in the ISAR imaging system, the narrowband tracking signals can be transmitted with a much lower rate, for the velocity tracking information is not needed to get focused range profiles. Therefore, we can achieve a higher wideband data rate with the HFM radar system, which is also significant for the ISAR imaging processing.

Although we concentrate on the situation for which the target moves with a constant velocity in this paper, the proposed method still works when the target has a constant acceleration. Additionally, the influence of the target’s acceleration can be easily compensated by a bank of filters with different frequency biases [7,19].

## Figures and Tables

**Figure A1:**
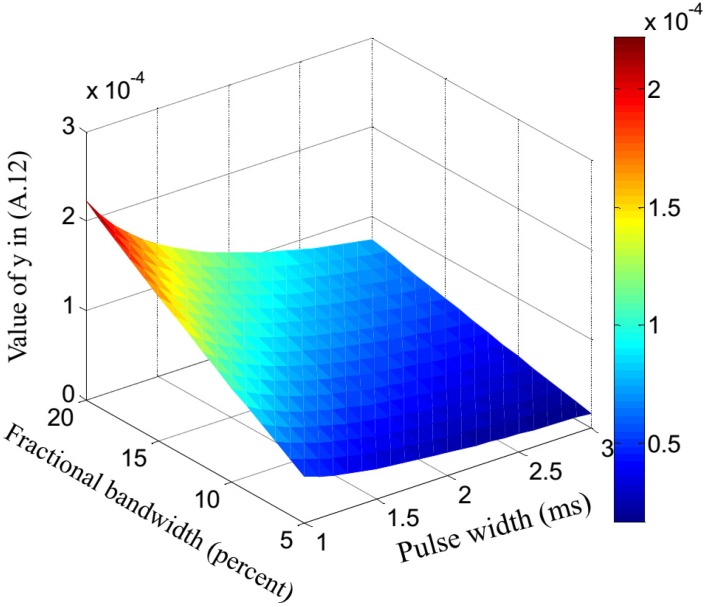
The value of *y* in Equation (A12).

**Figure A2:**
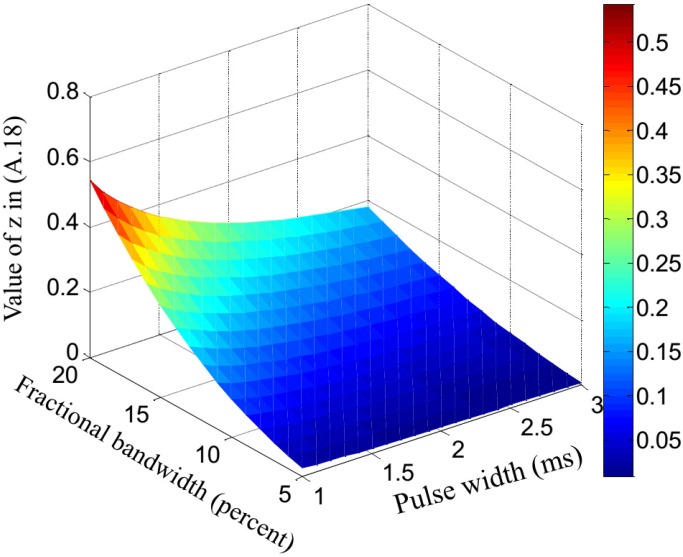
The value of *z* in Equation (A18).

## Appendix

### A. Simplification of the Logarithmic Phase in Equation (18)

The phase term of Equation (17) may be expanded based on the Taylor series of ln(1-x) as:

(A1) $$\varphi \left(t\right)=\frac{2\pi }{b}\ln\left[1-\frac{\gamma \tau_{mn}}{f_{c}-\gamma \left(t-\tau_{m}\right)}\right]=\sum_{i=1}^{\infty }\varphi_{i}\left(t\right)$$

where:

(A2) $$\varphi_{i}\left(t\right)=\frac{2\pi }{b}\frac{{\left(-1\right)}^{i}}{i}x^{i}\left(t\right)$$

and:

(A3) $$x\left(t\right)=\frac{\gamma \tau_{mn}}{f_{c}-\gamma \left(t-\tau_{m}\right)}$$

Next, we will prove that the components with i≥2 in Equation (A1) are all negligible in practice. If we set:

(A4) $$\varphi^{\prime }\left(t\right)=\frac{2\pi }{b}\sum_{i=2}^{\infty }\frac{{\left(-1\right)}^{i}}{i}x^{i}\left(t\right)$$

and:

(A5) $$s^{\prime }\left(t\right)=\exp\left(j\varphi^{\prime }\left(t\right)\right)$$

then the output of the decurve processing in Equation (17) can be rewritten as:

(A6) $$s_{hd}\left(t\right)=s_{1}\left(t\right)s^{\prime }\left(t\right)$$

where:

(A7) $$s_{1}\left(t\right)=\sigma_{n}a\left(\frac{t-\tau_{m}}{T_{p}}\right)\exp\left[j\varphi_{1}\left(t\right)\right]$$

The instantaneous frequency of $s^{\prime }\left(t\right)$ will be:

(A8) $$\begin{array}{c} f^{\prime }\left(t\right)=\frac{1}{2\pi }\frac{\partial \varphi^{\prime }\left(t\right)}{\partial t}=\frac{{\left(-1\right)}^{i}}{b}\sum_{i=2}^{\infty }x^{i-1}\left(t\right)\frac{\partial x\left(t\right)}{\partial t}\\ \;\;\;\;\;\;\;\;\;=\frac{{\left(-1\right)}^{i}}{b}\sum_{i=2}^{\infty }x^{i-1}\left(t\right)\frac{\gamma^{2}\tau_{mn}}{{\left[f_{c}-\gamma \left(t-\tau_{m}\right)\right]}^{2}}\\ \;\;\;\;\;\;\;\;\;=\frac{{\left(-1\right)}^{i}}{b}\frac{\gamma }{f_{c}-\gamma \left(t-\tau_{m}\right)}\sum_{i=2}^{\infty }x^{i}\left(t\right) \end{array}$$

For $\left|t-\tau_{m}\right|\leq T_{p}/2$, it is easy to get that:

(A9) $$\frac{\gamma }{f_{c}}\frac{1}{1+\frac{B}{2f_{c}}}\left|\tau_{mn}\right|\leq \left|x\left(t\right)\right|\leq \frac{\gamma }{f_{c}}\frac{1}{1-\frac{B}{2f_{c}}}\left|\tau_{mn}\right|$$

and:

(A10) $$\left|\tau_{mn}\right|\leq \frac{r_{wid}}{c}$$

where $r_{wid}$ is the maximum width of the imaging scene. If $r_{wid}$ is set to be no larger than 300 m, then:

(A11) $$\left|\tau_{mn}\right|\leq \frac{300m}{c}\approx 10^{-6}s$$

Substituting Equation (A11) into Equation (A9) yields:

(A12) $$0\leq \left|x\left(t\right)\right|\leq y=\frac{\gamma }{f_{c}}\frac{10^{-6}}{1-\frac{B}{2f_{c}}}$$

In this paper, we focus on fast moving target imaging with long-range distance, such as satellites and space debris. Therefore, we mainly are concerned with wideband signals with long pulse widths; here, we set the fractional bandwidth to satisfy:

(A13) $$0.05\leq \frac{B}{f_{c}}\leq 0.2$$

and the pulse width is:

(A14) $$T_{p}\geq 1ms$$

Substituting Equations (A13) and (A14) into Equation (A12), the value of *y* is shown in Figure A1. It is clear to see that:

(A15) $$\left|x\left(t\right)\right|<3\times 10^{-4}\ll 1$$

Considering the condition of Equation (A15), we obtain:

(A16) $$\begin{array}{c} \left|f^{\prime }\left(t\right)\right|\leq \frac{1}{\left|b\right|}\frac{\gamma }{f_{c}-\frac{B}{2}}\sum_{i=2}^{\infty }{\left|x\left(t\right)\right|}^{i}\\ \;\;\;\;\;\;\;\;\;\;=\frac{1}{\left|b\right|}\frac{\gamma }{f_{c}-\frac{B}{2}}\frac{{\left|x\left(t\right)\right|}^{2}}{1-\left|x\left(t\right)\right|} \end{array}$$

Substituting Equation (A12) into Equation (A16), we obtain:

(A17) $$\left|f^{\prime }\left(t\right)\right|\leq \frac{1}{\left|b\right|}\frac{\gamma }{f_{c}-\frac{B}{2}}\frac{y^{2}}{1-y}$$

For a waveform with pulse width $T_{p}$, the frequency resolution cell with FFT processing is $\Delta f=1/T_{p}$. Then, the ratio of $\left|f^{\prime }\left(t\right)\right|$ to Δf will be:

(A18) $$\frac{\left|f^{\prime }\left(t\right)\right|}{\Delta f}\leq z=\frac{1}{\left|b\right|}\frac{B}{f_{c}-\frac{B}{2}}\frac{y^{2}}{1-y}$$

For an X-band radar, if the carrier frequency is $f_{c}=10$ GHz, considering the parameters in Equations (A13) and (A14), the value of *z* is shown in Figure A2. We can see that z<0.6, which indicates that the maximum frequency of $s^{\prime }\left(t\right)$ is less than one frequency resolution cell, which also means that the components with i≥2 in Equation (A1) can be neglected. Therefore,

(A19) $$\begin{array}{c} \varphi \left(t\right)\approx \varphi_{1}\left(t\right)=-\frac{2\pi }{b}\frac{\gamma \tau_{mn}}{f_{c}-\gamma \left(t-\tau_{m}\right)}\\ \;\;\;\;\;\;\;\;=2\pi H\left[f_{c}\tau_{mn}+\frac{\gamma \tau_{mn}\left(t-\tau_{m}\right)}{1-\frac{\gamma }{f_{c}}\left(t-\tau_{m}\right)}\right] \end{array}$$

where $H=f_{L}f_{H}/f_{c}^{2}$ is a constant value. By differentiating Equation (A19), the instantaneous frequency of $s_{hd}\left(t\right)$ can be gotten as:

(A20) $$f_{hd}\left(t\right)=\frac{1}{2\pi }\frac{\partial \varphi \left(t\right)}{\partial t}=\frac{H\gamma \tau_{mn}}{{\left[1-\frac{\gamma }{f_{c}}\left(t-\tau_{m}\right)\right]}^{2}}$$

Taking the absolute value on both sides of Equation (A20), we have:

(A21) $$\begin{array}{c} \left|f_{hd}\left(t\right)\right|=\frac{H\gamma \left|\tau_{mn}\right|}{{\left|1-\frac{\gamma }{f_{c}}\left(t-\tau_{m}\right)\right|}^{2}}\leq \frac{H\gamma \left|\tau_{mn}\right|}{{\left|1-\frac{\gamma }{f_{c}}\frac{T_{p}}{2}\right|}^{2}}\\ \;\;\;\;\;\;\;\;\;\;\;\;\leq \frac{H\gamma }{{\left(1-\frac{B}{2f_{c}}\right)}^{2}}\frac{r_{wid}}{c} \end{array}$$

Therefore, the bandwidth of $s_{hd}\left(t\right)$ will be:

(A22) $$B_{d}=2\times \max\left|f_{hd}\left(t\right)\right|\leq H\gamma \frac{2r_{wid}}{c}\frac{1}{{\left(1-\frac{B}{2f_{c}}\right)}^{2}}$$
